# A Feature Engineering Method for Smartphone-Based Fall Detection

**DOI:** 10.3390/s25206500

**Published:** 2025-10-21

**Authors:** Pengyu Guo, Masaya Nakayama

**Affiliations:** 1 Department of Electronic Engineering and Information Systems, The University of Tokyo, Tokyo 113-8654, Japan; kaku-houu@g.ecc.u-tokyo.ac.jp; 2 Information Technology Center, The University of Tokyo, Tokyo 113-8654, Japan

**Keywords:** fall detection, smartphone, human motion recognition, feature engineering, interpretability analysis

## Abstract

A fall is defined as an event in which a person inadvertently comes to rest on the ground, floor, or another lower level. It is the second leading cause of unintentional death worldwide, with the elderly population (aged 65 and above) at the highest risk. In addition to preventing falls, timely and accurate detection is crucial to enable effective treatment and reduce potential injury. In this work, we propose a smartphone-based method for fall detection, employing K-Nearest Neighbors (KNN) and Support Vector Machine (SVM) classifiers to predict fall events from accelerometer data. We evaluated the proposed method on two simulated datasets (UniMiB SHAR and MobiAct) and one real-world fall dataset (FARSEEING), performing both same-dataset and cross-dataset evaluations. In same-dataset evaluation on UniMiB SHAR, the method achieved an average accuracy of 98.45% in Leave-One-Subject-Out (LOSO) cross-validation. On MobiAct, it achieved a peak accuracy of 99.89% using KNN. In cross-dataset validation on MobiAct, the highest accuracy reached 96.41%, while on FARSEEING, the method achieved 95.35% sensitivity and 98.12% specificity. SHAP-based interpretability analysis was further conducted to identify the most influential features and provide insights into the model’s decision-making process. These results demonstrate the high effectiveness, robustness, and transparency of the proposed approach in detecting falls across different datasets and scenarios.

## 1. Introduction

A fall is defined as an event which results in a person coming to rest inadvertently on the ground or the floor or other lower level [1]. And a fall detection system (FDS) is an assistive device whose main objective is to detect and alert when a fall happens. Falls may lead to serious injuries such as bones fracture, bruise, internal bleeding, or even death [2]. In fact, falls are the second leading cause of unintentional injury death according to the World Health Organization (WHO), with the elderly population (aged 65 and above) being at the highest risk [1].

Moreover, population aging is a prominent problem globally. According to the World Bank, in 2024, the world aging rate is 10%, with 30% in Japan, and 15% in China with a rapid growth trend.

According to the Centers for Disease Control and Prevention (CDC) of the United States, in 2018, 27.5% adults aged 65 and above reported falling in the past year, which results in 36 million falls [3]. Among these falls, 10.2% caused injuries, which indicates 8.4 million fall-related injuries [3]. Meanwhile, in 2021, the age-adjusted fall death rate is 78.0 per 100,000 older adults, which has increased by 41% compared with that in 2012 [4]. Similarly, the Consumer Affairs Agency of Japan reported that the number of deaths among the elderly due to “falls, tumbles, and crashes” is about 4 times that of “traffic accidents” [5].While causing casualties, falls caused huge financial loss. Florence CS, et al. [6] concluded that the estimated medical cost attributed to falls was approximately USD 50 billion in 2015. For fatal falls, the cost was estimated to be USD 754 million. Additionally, the medical outcome of a fall is largely depending on the response and rescue time [7]. When a fall happens, timely rescue can help prevent the injury from being worsened, also helping to reduce medical costs. All these facts indicate that an FDS is needed.

**Figure 1:**
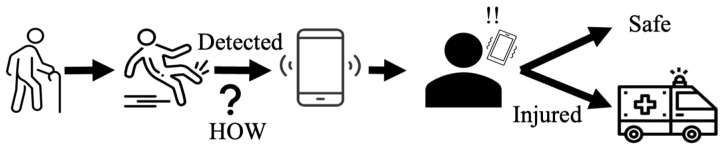
Smartphone-based Fall Detection Model.

Researchers have proposed some FDSs, which can be divided into four categories [8]—vision- based, ambient device-based, wireless signal-based, and wearable sensor-based. The smartphone-based approach is one of the wearable sensor-based solutions. We compared these solutions in Section 2. The process of smartphone based fall detection system is shown in Figure 1.

We are focusing on the smartphone-based approach because (1) There are multiple sensors planted in the smartphone, which can provide data for fall detection; (2) Although it requires users to carry smartphone, it is environment free—it can detect falls anywhere if only the smartphone is carried, and it is not affected by other people or items around; (3) The smartphone is widely used and users do not need to carry additional devices; (4) The smartphone is a communication device, which makes it easy to contact others when a fall is detected.

In this work, we propose a fall detection algorithm employing data from a built-in smartphone accelerometer. The main contributions are as follows:

1. We propose a novel smartphone-based fall detection algorithm, named *3s2sub*, which divides a 3 s motion window into two overlapping sub-windows to extract multiple statistical features for robust classification.
2. We conduct preliminary experiments to quantify the effect of key design parameters—including window length, sub-window number, and overlap ratio—and confirm the optimality of the chosen configuration (3 s window, two sub-windows, 50% overlap).
3. We conduct an evaluation of *3s2sub* on a single-event dataset (UniMiB SHAR), achieving an average detection accuracy of over 98% in Leave-One-Subject-Out (LOSO) cross-validation.
4. We evaluate *3s2sub* on a lab-simulated continuous-event dataset (MobiAct), performing both same-dataset evaluation (train and test on MobiAct) and cross-dataset evaluation (train on UniMiB SHAR, test on MobiAct). In the same-dataset evaluation, the proposed method achieves a peak accuracy of 99.84%, while in the cross-dataset evaluation, it achieves a peak accuracy of 96.41%.
5. We evaluate *3s2sub* on a real-world fall dataset (FARSEEING) in a cross-dataset setting (train on UniMiB SHAR, test on FARSEEING), achieving 95.35% sensitivity (an improvement of 2.02–8.68% over existing works) and 98.17% specificity (an improvement of 11.12–24.29% over existing works).
6. We employ SHAP-based interpretability analysis to identify the most influential features and reveal their interactions, thereby providing insights into the model’s decision-making process and enhancing the transparency of the proposed method.

The remainder of this paper is organized as follows. Section 2 reviews related work, covering both non-smartphone-based and smartphone-based approaches. Section 3 details the proposed methodology, including feature extraction, classification, and performance evaluation. Section 4 introduces the three datasets used in this study. Section 5 presents the results of preliminary experiments, which justify the selection of each hyperparameter. Section 6 reports the experimental setup and results, encompassing both single-event and continuous-event dataset evaluations. Section 7 provides the SHAP-based interpretability analysis, quantifying the contribution of individual features and offering insights into the model’s decision-making process. Section 8 discusses the results in the context of existing literature. Finally, Section 9 concludes the paper by summarizing its contributions and limitations, and by outlining potential directions for future research.

## 2. Related Work

### 2.1. Traditional Machine Learning Approaches

Traditional fall detection methods often employ hand-engineered features extracted from smartphone sensors, combined with classical machine learning algorithms such as threshold-based methods, SVMs, decision trees, or simple ensemble classifiers.

S. Nooruddin et al. [9] proposed an IoT-based FDS that is device-independent, working with smartphones or other sensor-equipped platforms like Raspberry Pi. They used a linear classifier and achieved 99% accuracy, 96.3% sensitivity, 99.2% precision, and 97.7% F1 score. D. Morozek et al. [10] utilized Boosted Decision Trees trained on data from smartphone accelerometer and gyroscope, achieving 99.9% accuracy and 100% precision on the SisFall [11] dataset. J.-S. Lee et al. [12] developed a threshold-based method using signal magnitude area and axis-wise mean acceleration values, attaining 99.38% accuracy. M. Soni et al. [13] proposed a fog computing-based FDS using SVM with a custom kernel function and five time-domain features, achieving 99.25% accuracy, 99.53% F1 score, and 100% sensitivity. J. C. Dogan et al. [14] introduced a two-stage fall detection framework, converting multi-class outputs from various tri-axial sensors (accelerometer, gyroscope, etc.) into binary decisions. They achieved 95.65% accuracy using gyroscope and SVM. De Quadros T. et al. [15] proposed a machine learning based FDS with a wrist-worn IMU, which includes a tri-axial accelometer, a tri-axial gyroscope, and a tri-axial magnetometer. It achieved 99% accuracy and 100% sensitivity. D. Giuffrida et al. [16] a supervised machine learning-based fall detection method, which used data from a tri-axial accelerometer and gyroscope. They slice the signal into windows and extract features, including maximum, minimum, and slice frequency bins. With a SVM, they achieved F1 Score over 97% and sensitivity over 99.7%.

Some studies also combine smartphones with other wearables. T. Vilarinho et al. [17] proposed a threshold-based method using both smartphone and smartwatch data, achieving 63% fall detection rate and 78% ADL recognition. S.-L. Hsieh et al. [18] combined wristband devices and smartphones with a two-step thresholding method, reaching 100% peak accuracy.

### 2.2. Deep Learning-Based Approaches

Recent work has increasingly adopted deep learning architectures to learn representations directly from raw sensor data. These methods, while often achieving high accuracy, tend to be computationally expensive and less interpretable.

M. M. Hassan et al. [19] introduced a smartphone-based FDS using a deep CNN-LSTM module trained on the MobiAct [20] dataset, reaching 96.75% accuracy. Choi A et al. [21] proposed a near- FDS, which is based on a waist-worn IMU and deep learning. With features that are extracted from data of accelerometer and gyroscope, the algorithm achieved accuracy of over 98%. X. Wu et al. [22] employed a GRU-based model and reported 99.39% accuracy and 95.06% F1 score. X. Qian et al. [23] designed a distributed deep CNN hierarchy for fall detection, achieving 99.70% accuracy. D. Mukherjee et al. [24] proposed an ensemble deep learning model for fall detection, which used CNN-Net, Encoded-Net, and CNNLSTM models to vote. The best accuracy is reported to 99.7%.

Recent studies (2024–2025) further explored hybrid architectures and embedded deep learning for fall detection. Al-qaness et al. [25] proposed a CNN–Transformer hybrid model for human activity recognition and fall detection, demonstrating strong performance on public datasets, F1 score of 98.30% on UniMiB SHAR. Campanella et al. [26] presented an embedded deep learning sensor system for wearable fall detection, achieving real-time classification with low-latency performance on edge devices, which achieved peak accuracy of 99.38% and inference time of 25 ms. Another work by Al-Qaness et al. [27] proposed MKLS-Net, combining multi-kernel convolution, LSTM, and self-attention to capture both spatial and temporal features. The method demonstrated high accuracy on wearable sensor data, but with relatively high computational demands. The accuracy is reported to be 99.51% and 99.40% on MobiAct and UniMiB SHAR.

### 2.3. Summary

Although these existing models have demonstrated promising results, several limitations remain:

1. Many rely on deep learning architectures [19,22,23], which typically require substantial computational resources and may not be suitable for deployment on smartphones.
2. Most methods are evaluated only on a single dataset, limiting their ability to generalize to unseen users or environments.
3. Many works are conducted on simulated datasets collected in controlled lab settings, which may not reflect the complexity of real-world falls.
4. Deep models often function as black boxes, providing limited interpretability.

In contrast, our work proposes a lightweight, interpretable, and generalizable alternative that addresses these challenges.

## 3. Methods

As discussed in Section 2, existing fall detection methods face several limitations: (1) reliance on computationally expensive deep learning models, (2) training and evaluation on a single dataset, (3) exclusive use of simulated laboratory data, and (4) limited interpretability of model decisions.

To address these issues, we propose a feature engineering-based fall detection approach, referred to as *3s2sub*.

(1) Instead of relying on deep learning, our method employs lightweight statistical and temporal features in conjunction with traditional machine learning models (KNN and SVM), enabling efficient computation on resource-constrained devices such as smartphones. (2–3) We conduct a comprehensive evaluation on three datasets covering single-event simulated, continuous-activity simulated, and real-world fall scenarios. The experiments include both in-dataset and cross-dataset validation to assess generalization ability. (4) We further perform a SHAP [28]-based interpretability analysis to provide insights into the decision-making process of the proposed method.

### 3.1. Data Segmentation

After analyzing the raw data, we observed that the duration of a typical fall generally ranges from 1 to 3 s, with the most abrupt changes occurring at the *beginning* and *ending* of the fall. These changes provide discriminative cues for distinguishing falls from other activities.

Based on this observation, we hypothesize that a fall event can be divided into two distinct stages—“beginning” and “ending”—represented by two sub-windows, and that features should be extracted from each sub-window separately. We denote a window of length *n* seconds with two sub-windows as ***n**2sub*.

Under this hypothesis, three hyperparameters are subject to fine-tuning:

1. **Window length** (*n*): The window should be long enough to capture the complete fall event while avoiding unnecessary background activities.
2. **Overlap ratio** (*o*) between sub-windows.
3. **Number of sub-windows** (*m*): Whether the fall event should be divided into more stages (e.g., “beginning”, “early middle”, “late middle”, “ending”).

**Figure 2:**
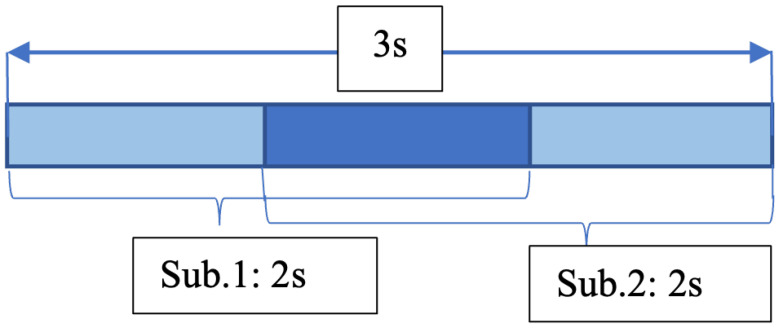
3s2sub Data Segmentation. Window size is set to three seconds. Each window is divided into two sub-windows, whose length are two seconds. The overlap of the sub-windows is 50% (one second).

We conducted preliminary experiments to determine the optimal hyperparameters (details in Section 5). The results indicated that *3s2sub* achieved the best performance. As shown in Figure 2, the final configuration uses a 3 s window divided into two sub-windows with a 50% overlap, from which features are extracted separately.

### 3.2. Feature Extraction

In this work, we use accelerometer data, which is sampled at 50 Hz. As a result, each action event is represented by a 150×3-dimensional matrix, corresponding to the three measurement axes.

We extracted eight features from the two sub-windows separately, as detailed in Table 1. Mean represents the overall level of the data. The median provides a better representation of a ‘typical’ value, since it would not be skewed by a small proportion of extreme large or small values. Standard deviation (SD) measures the distribution, in other words, a low SD refers to the acceleration tending to be close to the mean, while a high SD refers to the acceleration distributed in a wider range. A negative skew means the majority of the samples has high values, while the higher kurtosis indicates that there are more outliers. The maximum and minimum represent the extreme values. These features are all statistical and include time domain features, for which the calculation complexity is very low.

Given an input window $X=\{x_{1},x_{2},\ldots ,x_{n}\}$, we define the extracted features as

- Mean:
  (1) $$\mu =\frac{1}{n}\sum_{i=1}^{n}x_{i}$$
- Median:
  (2) $$\mathrm{median}\left(X\right)=\left\{\begin{array}{cc} x_{\left(\frac{n+1}{2}\right)}, & \mathrm{if}\;n\;\mathrm{is}\;\mathrm{odd}\\ \frac{1}{2}\left(x_{\left(\frac{n}{2}\right)}+x_{\left(\frac{n}{2}+1\right)}\right), & \mathrm{if}\;n\;\mathrm{is}\;\mathrm{even} \end{array}\right.$$
- Standard Deviation:
  (3) $$\sigma =\sqrt{\frac{1}{n}\sum_{i=1}^{n}{\left(x_{i}-\mu \right)}^{2}}$$
- Skewness:
  (4) $$\mathrm{Skew}=\frac{\frac{1}{n}\sum {\left(x_{i}-\mu \right)}^{3}}{\sigma^{3}}$$
- Kurtosis:
  (5) $$\mathrm{Kurt}=\frac{\frac{1}{n}\sum {\left(x_{i}-\mu \right)}^{4}}{\sigma^{4}}$$
- Maximum/Minimum: max(X),min(X)
- Slop:
  (6) $$SL=\sqrt{{\left(max_{x}-min_{x}\right)}^{2}+{\left(max_{y}-min_{y}\right)}^{2}+{\left(max_{z}-min_{z}\right)}^{2}}$$

As is known, an accelerometer has three axes (x, y, z). The feature 1–7 is calculated by axis. The slop is calculated by data from all three axes, as Equation (6) shows. The features extracted from each axes are integrated into a row vector. Concretely, for each activity sample, the feature vector is a 1 × 44-dimensional vector.

### 3.3. Classifier

The feature vector is then input into classifiers for prediction. In this work, we employed two classifiers for comparison: SVM and K Nearest Neighbor (KNN).

SVM is known as a binary classifier, which is suitable to distinguish falls and ADLs. It is utilized in various related works [13,14,29]. We used SVM with a Gaussian Kernel. Given a set of training data, each of them is labelled belonging to one of the two classes. SVM builds a model to map the training examples to space as “points”, and find a hyperplane, which makes the distance between the points and the hyperplane is maximized.

The processing of KNN is always as follows: (1) calculate the distance ($D_{x_{i}y}$) between the training samples ($x_{i}$) and the predicting samples (y), (2) sort $D_{x_{i}y}$, (3) select K points which have the shortest $D_{x_{i}y}$, (4) label the predicting sample as the K points’ label. KNN is easy to implement and interpret because it is simply based on the calculation between the samples [30].

We conducted hyperparameter selection for both classifiers through 10-fold cross-validation on the training set of each dataset: For Support Vector Machine (SVM), we compared four kernel functions: linear, Gaussian (RBF), sigmoid, and polynomial. Among them, the Gaussian kernel consistently achieved the best performance across all datasets. Therefore, we adopted the RBF kernel with γ = scale, which automatically sets $\gamma =\frac{1}{n_{\mathrm{features}}\cdot \mathrm{Var}\left(X\right)}$.

  For K-Nearest Neighbors (KNN), we performed a grid search over k∈{1,2,3,4,5}. The best performance was consistently obtained with k=1, which we adopted for all reported results.

These parameter settings were fixed throughout all same-dataset and cross-dataset experiments for consistency and fairness.

### 3.4. Evaluation Metrics

We employed several evaluation metrics to assess our model, including accuracy, Macro Average Accuracy [29], precision, sensitivity, and F1 Score. These metrics help quantify the model’s performance across various dimensions.

Firstly, some relevant abbreviations are defined as

**True Positive (TP)**: Samples that are predicted as *Fall* whose true labels are *Falls*.

**True Negative (TN)**: Samples that are predicted as *ADL* whose true labels are *ADLs*.

**False Positive (FP)**: Samples that are predicted as *Fall* whose true labels are *ADLs*.

**False Negative (FN)**: Samples that are predicted as *ADL* whose true labels are *Falls*.

With these abbreviations, the detailed definitions and explanations of each metric are as follows:

- **Accuracy**: The proportion of the samples are predicted correctly out of the total samples, defined as Equation (7). It evaluates the overall performance of the model.
  (7) $$Accuracy=\frac{TN+TP}{TN+TP+FN+FP}$$
- **MAA**: Given *E* the set of all the activity types, a∈E, $T_{a}$ the total number of times that *a* occurs in the dataset; $TP_{a}$ the number of times the activity *a* is predicted correctly, MAA (*Macro Average Accuracy*) is defined by Equation (8) [29]. MAA is the arithmetic average of the accuracy $Acc_{a}$ of each activity. It allows each partial accuracy to contribute equally to the evaluation [29].
  (8) $$MAA=\frac{1}{\left|E\right|}\sum_{a=1}^{\left|E\right|}Acc_{a}=\frac{1}{\left|E\right|}\sum_{a=1}^{\left|E\right|}\frac{TP_{a}}{T_{a}}$$
- **Sensitivity**: Sensitivity explains how many positive samples are predicted correctly, as defined as (9). In the case of FDS, it refers to how many falls are ‘discovered’. FDS is a system that users entrust their lived to, for which it is very important to make sure as many falls as possible to be ‘discovered’. Thus, we consider sensitivity as a important indicator to evaluate FDS.
  (9) $$Sensitivity=\frac{TP}{TP+FN}$$
- **F1 Score**: F1 score evaluates the predictive skill of the model, which combine precision and sensitivity. Compared with accuracy, F1 score elaborates on class-wise performance, while accuracy focus on overall performance.
  (10) $$F1Score=2\times \frac{Sensitivity\times Precision}{Sensitivity+Precision}$$
- **Specificity**: Specificity measures the proportion of actual negatives that are correctly identified by the model. In the case of fall detection, higher specificity refers to less false alarm.
  (11) $$Specificity=\frac{TN}{TN+FP}$$
- **Precision**: Precision explains how many true positive samples. In the case of fall detection, higher precision refers to less false alarm. It is easy to identify that precision and sensitivity are competing metrics.
  (12) $$Precision=\frac{TP}{TP+FP}$$

## 4. Datasets

There are more than 20 fall datasets that have been published. We have compared some fall datasets and have selected three datasets—UniMiB SHAR [29], MobiAct [20], and FARSEEING [31] to perform experiments. We selected these datasets because: (1) UniMiB SHAR and MobiAct contains data from more subjects. (2) UniMiB SHAR and MobiAct are collected by smartphones. (3) FARSEEING contains real-world fall records from the elderly. These datasets are all publicly available.

### 4.1. UniMiB SHAR

UniMiB SHAR [29] was published in 2016. It comprises 11,771 activity samples (7579 ADLs and 4192 falls) of 17 types of activity (8 falls and 9 ADLs), which were collected from 30 subjects (24 women and 6 men), aged from 18 to 60. In this dataset, only acceleration (x, y, z axis) was collected, in the range of ±2g. All data in this dataset are sampled at 50 Hz for 3 s. Because the data are all for 3 s and included only one kind of activity in each sample, we call it single-event dataset.

### 4.2. MobiAct

The MobiAct [20] dataset was published in 2016. The authors collected data with a smartphone, with 66 participants, aged from 20 to 47, performing 16 types of activities, including 4 types of falls and 12 types of ADLs. They collected acceleration (x, y, z axis), angular velocity (x, y, z axis), and orientation (Azimuth, Pitch, Roll). Acceleration is in range of ±2g. There are 3199 samples, including 767 falls and 2432 ADLs. In this dataset, falls are collected for 10 s, and ADLs are collected in different lengths (6 s to 5 min). We treat it as a continuous-event dataset.

### 4.3. FARSEEING

**Figure 3:**
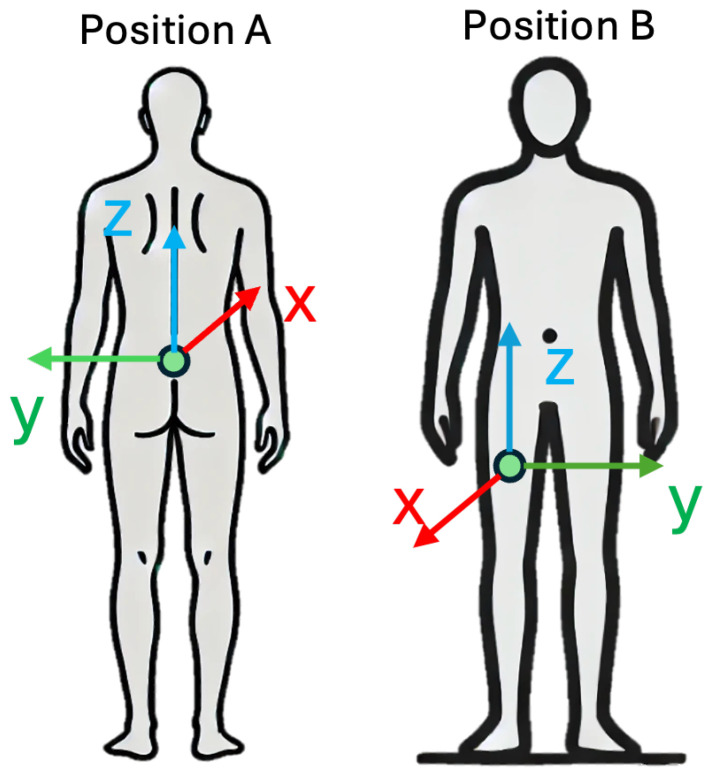
FARSEEING dataset sensor position.

FARSEEING [31] is a dataset that contains real-world falls. The authors claimed that they have collected more than 300 real-world falls from January 2012 to December 2015. Unfortunately, there are only 22 verified falls records that have been released. These data were collected from 15 subjects, aged from 56 to 86. The data were collected in a rehabilitation clinic or at home. The sensors were located at lower back (Position A) or thigh (Position B), as shown in Figure 3. They used two types of devices to collect acceleration data, which results in data collected in two ranges—±2g and ±6g. Concretely, 7 examples are in ±6g and 15 are in ±2g.

We used all 22 examples to evaluate our method. These were collected from 15 subjects (8 women and 7 men). Among these 22 examples, 15 were collected from Position A, 7 were collected from Position B. Position A records are sampled at 100 Hz, and Position B records were sampled at 20 Hz. Although the devices are not put in trousers pocket (as in UniMiB SHAR and MobiAct), we think it is feasible to evaluate our method because Position A corresponds to a shallow trouser pocket, while position B corresponds to a very large trouser pocket. All data in FARSEEING are 1200 s long.

Because our method *3s2sub* assumes each ‘event’ last for 3 s, we consider both MobiAct and FARSEEING as continuous-event datasets.

## 5. Preliminary Experiments

The preliminary experiments were conducted to quantify the contribution of each key design choice—namely window length, sub-window number, and sub-window overlap ratio (sub-window length)—to the overall performance. All reported results are based on 10-fold cross-validation.

**Figure 4:**
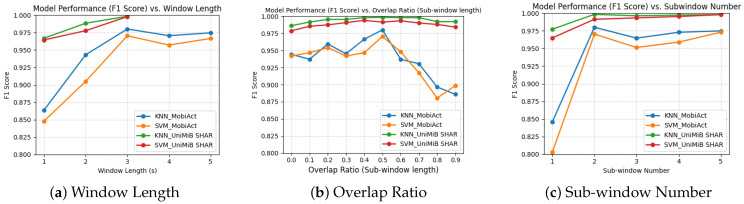
Preliminary Results (**a**): ***n**s2sub*, n∈[1,5] (**b**) *3s2sub*, overlap between two sub-windows *o*, o∈[0.1,0.9], o increases by 0.1 (**c**) *3s**m**sub*, m∈[1,5].

First, we evaluated ***n**s2sub* with n∈[1,5], where *n* denotes the window length in seconds. Although the window length could take various values (e.g., 2.2, 2.8, etc.), we selected integer values for simplicity and ease of processing (Figure 4a) For both datasets, shorter windows (1–2 s) led to a 3–10% drop in F1 score, likely due to incomplete capture of the fall process. Conversely, longer windows (4–5 s) caused a moderate decrease (1–2%), possibly due to the inclusion of irrelevant motion data, which diluted the discriminative features.

Second, we examined *3s2sub* with varying overlap ratios o∈[0,0.9] in increments of 0.1 Figure 4b). For both datasets, an overlap of o=0.5 (50%) yielded the highest F1 scores, indicating that this configuration provides optimal coverage of both the “beginning” and “ending” phases of a fall, while avoiding excessive redundancy.

Finally, we tested *3s**m**sub* with m∈[1,5], where *m* is the number of sub-windows (Figure 4c). The trends were consistent across datasets and classifiers (KNN, SVM), with m=2 achieving the best performance. This suggests that dividing each window into two overlapping sub-windows strikes an effective balance between temporal resolution and contextual coverage.

## 6. Experiment & Results

To evaluate *3s2sub*, we conducted experiments in two parts: an evaluation on a single-event dataset (UniMiB SHAR) and an evaluation on continuous event data (MobiAct and FARSEEING).

### 6.1. Evaluation on Single-Event Data Set

The evaluation was performed using UniMiB SHAR [29].

The authors of UniMiB SHAR provided an evaluation for the dataset. We designed experiments with reference to the evaluation, and performed a 5-Fold Cross-Validation and LOSO Cross-Validation, which were performed in [29]. The 5-Fold Cross-Validation involves randomly splitting the dataset into five parts. In each iteration, one fold is considered as a test fold, while the others are considered as training folds. LOSO Cross-Validation is to test the classifier on data from unseen subjects. This is to test the performance subject-independently. Additionally, we conducted a 30-Fold Cross-Validation as a control group to compare the results of LOSO Cross-Validation. We split the data randomly into 30 splits, and considered one as the test fold in each iteration and the others as training folds. Because the dataset is imbalanced, we applied the synthetic minority oversampling technique (SMOTE) in the training phase to balance it.

Also, in each cross-validation, we set four control groups with different feature vectors—*2s1*, *2s2*, *3s*, and *Raw Data*. All experiments were performed in MATLAB R2020b using a MacBook Air 2020 equipped with an Apple M1 chip.

- *2s1*: a 22-dimensional feature vector extracting the same features (mean, median, standard deviation, skew, kurtosis, maximum, minimum, slope) by axes from the first sub-window (Sub1).
- *2s2*: a 22-dimensional feature vector extracting the same features by axes from the second sub-window (Sub2).
- *3s*: a 22-dimensional feature vector extracting the same features by axes from the whole *3s* window, without separating into sub-windows.
- *Raw Data*: a 453-dimensional feature vector of data without any processing.

**Figure 5:**
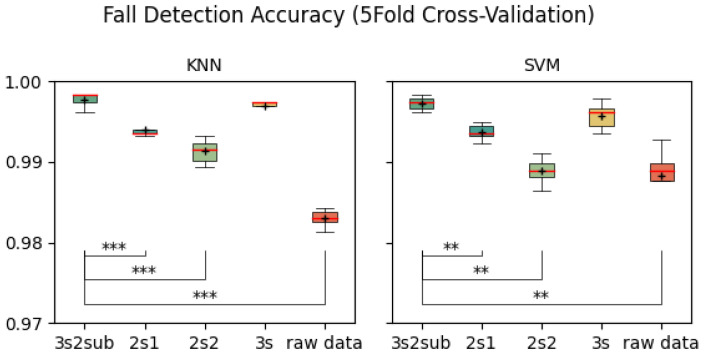
Fall detection accuracy (5-Fold Cross-Validation). **:p<0.01, ***:p<0.001, +: Mean value. The five parallel lines in box plot corresponding to (bottom to top) minimum, first quartile (the median of the lower half of the data), median (the red line here), third quartile (the median of the upper half of the data), and maximum.

**Figure 6:**
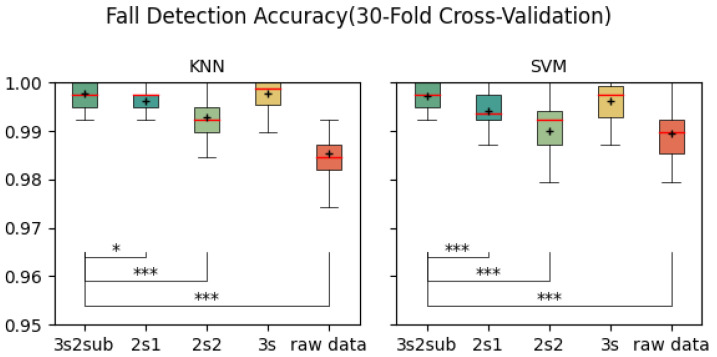
Fall detection accuracy (30-Fold Cross-Validation). *:p<0.05, ***:p<0.001, +: Mean value.

**Figure 7:**
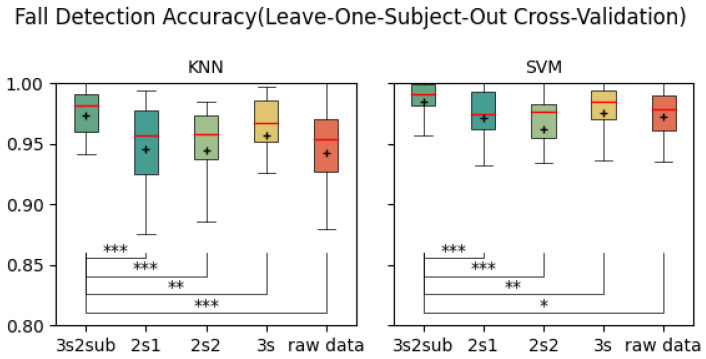
Fall detection accuracy (Leave-One-Subject-Out Cross-Validation). *:p<0.05, **:p<0.01, ***:p<0.001, +: Mean value.

Table 2 shows the average accuracy of each control group. As is shown, 3s2sub achieved the highest accuracy of 99.77% with KNN in the 5-Fold Cross-Validation, 99.78% in the 30-Fold Cross-Validation, and 98.45% with SVM in the LOSO Cross-Validation. Figure 5, Figure 6 and Figure 7 are the box plots of fall detection, including T-test results between *3s2sub* and other groups, in which “+” represents the mean value of each group. In 5-Fold and 30-Fold Cross-Validation, *3s2sub* showed significant difference from the *2s1*, *2s2*, and *Raw Data* groups with both KNN and SVM, while, in the LOSO Cross-Validation, *3s2sub* showed significant difference from the *2s1*, *2s2*, *3s*, and *Raw Data* groups, with both KNN and SVM.

**Figure 8:**
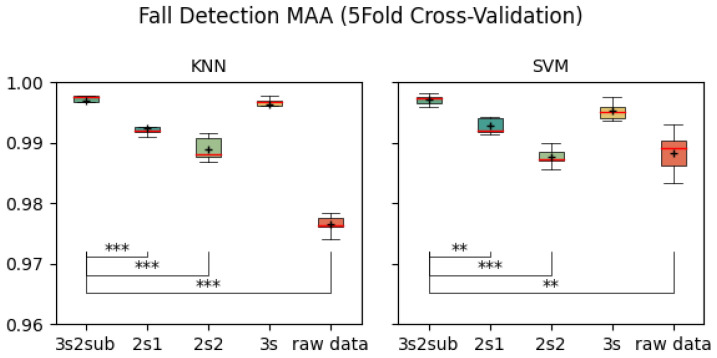
Fall detection MAA (5-Fold Cross-Validation). **:p<0.01, ***:p<0.001, +: Mean value.

**Figure 9:**
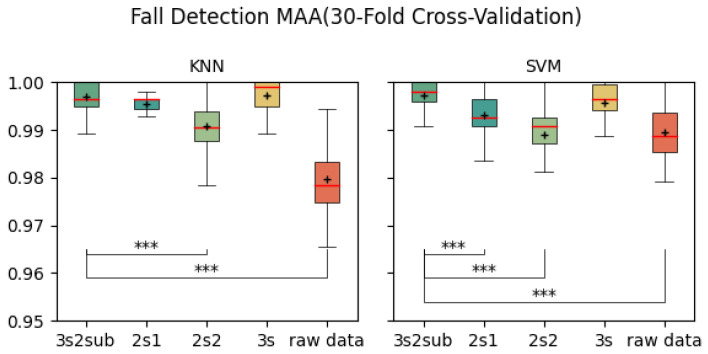
Fall detection MAA (30-Fold Cross-Validation). ***:p<0.001, +: Mean value.

**Figure 10:**
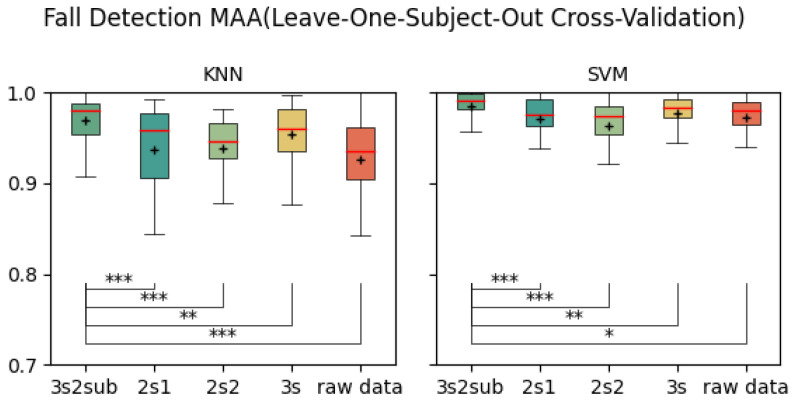
Fall detection accuracy (Leave-One-Subject-Out Cross-Validation). *:p<0.05, **:p<0.01, ***:p<0.001, +: Mean value.

Table 3 shows the average Macro Average Accuracy (MAA) of each control group. As is shown, 3s2sub achieved best result of 99.71% in 5-Fold Cross-Validation, 99.72% with SVM in 30-Fold Cross-Validation, and 98.48% in LOSO Cross-validation. Figure 8, Figure 9 and Figure 10 are the box-plots including T-test results between *3s2sub* and other control groups. Same as accuracy, ‘+’ represents the mean value of each group. In 5-Fold Cross-validation, *3s2sub* showed significant difference with 2s1, 2s2, and Raw Data group with both KNN and SVM. In 30-Fold Cross-Validation, *3s2sub* showed significant difference with *2s2* and Raw Data group with KNN, and with *2s1*, *2s2*, and Raw Data group with SVM. In LOSO Cross-Validation with *2s1*, *2s2*, *3s*, and Raw Data group with both KNN and SVM.

In addition, Table 4, Table 5 and Table 6 summarize the sensitivity, precision, and F1-score of all tested groups using KNN and SVM in 5-fold, 30-fold, and LOSO cross-validation, respectively.

As shown in Table 4, *3s2sub* achieved the highest sensitivity, precision, and F1-score among all groups in 5-fold cross-validation, with a sensitivity of 99.93%, precision of 99.74%, and F1-score of 99.84% using KNN, and a sensitivity of 99.75%, precision of 99.82%, and F1-score of 99.78% using SVM.

In 30-fold cross-validation (Table 5), *3s2sub* likewise obtained the highest results, achieving 99.96% sensitivity, 99.70% precision, and 99.83% F1-score with KNN, and 99.78% sensitivity, 99.82% precision, and 99.79% F1-score with SVM.

For LOSO cross-validation (Table 6), *3s2sub* achieved a sensitivity of 98.31%, precision of 97.59%, and F1-score of 97.89% with KNN, all of which were the highest among the tested groups. It also attained the highest SVM results, with 98.15% sensitivity, 99.35% precision, and 98.68% F1-score.

### 6.2. Evaluation on Continuous Dataset (MobiAct)

We performed same-dataset validation and cross-dataset validation with MobiAct. Since the sampling rate of MobiAct is 87 Hz (for accelerometer data), in both same-dataset and cross-dataset validation we performed down sampling. The data were down-sampled to 50 Hz. To facilitate model training, we need to segment the long-term data into 3 s segments. We used data annotated by the MobiAct authors. Finally, 767 falls and 18,239 ADLs are generated. Because it is highly imbalanced, we applied the synthetic minority oversampling technique (SMOTE) in the training phase to balance it. We performed a 10-fold cross-validation.

Table 7 shows accuracy, sensitivity, F1 Score, and specificity of KNN and SVM. For KNN classification, it achieved an accuracy of 99.84%, a sensitivity of 98.02%, an F1 Score of 98.01% and a specificity of 99.92%. For SVM classification, it achieved an accuracy of 99.76%, a sensitivity of 96.98%, an F1 Score of 97.04%, and a specificity of 99.88%.

Considering that we cannot collect data on all patterns of falls and ADLs, we designed cross-dataset validation to evaluate scalability and generalization. We first performed cross-dataset validation on two simulated datasets (UniMiB SHAR and MobiAct). Because real-word data are continuous, we used UniMiB SHAR as a training set and MobiAct as a test set.

For each sample in MobiAct, a slide window was applied for 3 s and a 50% overlap was applied, and *3s2sub* characteristics were extracted from each window. Then, the feature vectors were fed to pre-trained models (trained on UniMiB SHAR). The models output a label for each window. If there is a “fall” window detected in a sample, it outputs the label “Fall” for the sample; otherwise, the output label for the sample is “ADL.”

Table 8 shows the accuracy, sensitivity, F1 Score, and specificity of KNN and SVM. For classification using KNN, it achieved an accuracy of 96.41%, sensitivity of 92.05%, F1 score of 92.47%, and specificity of 97.78%. For classification using SVM, as shown in Table 8, it achieved an accuracy of 88.22%, sensitivity of 97.26%, F1 score of 79.83%, and specificity of 85.36%.

### 6.3. Head-to-Head Comparison

In Section 6, we showed the experiment results of our method. To perform a fairer comparison, we deployed two light-weight deep learning model—a vanilla 1D-CNN and a hybrid CNN-BiGRU [25] to compare with our method. We performed 10-fold cross-validation in this part.

The vanilla CNN (CNN base) comprises two convolutional layers with kernel sizes of 3 and 64 filter sizes. After the pooling operation, the output of the convolutional layer is fed to a fully connected layer with ReLU activation function and dimension 256. The CNN BiGRU-ATT network is similar to the CNN base network but with the addition of a third convolution layer and two BiGRU layers with attention and dimension 128. The output of the BiGRU with attention layers is then passed through a fully connected layer with the ReLU activation function and dimension 128. We deployed these two baselines following [25]. The results are detailed in Table 9.

To quantitatively substantiate our rationale for employing traditional machine learning models over deep learning alternatives, we conducted a comprehensive computational complexity analysis. This analysis directly addresses the critical requirements of real-time performance and resource efficiency for deployment on smartphone devices. The experiments were performed on a MacBook Air (2021) equipped with an Apple M1 CPU. The inference time reported encompasses both the feature calculation of our 3s2sub method and the final classification step. The results are detailed in Table 10.

The analysis reveals several key insights into the trade-offs between different modeling approaches:

1. **Inference Efficiency:** As illustrated in Table 10, our proposed method, particularly when paired with the KNN classifier, achieves the shortest inference time on both datasets (2.63 ms on MobiAct and 2.61 ms on UniMiB SHAR). This superior speed underscores its suitability for real-time, window-by-window processing on mobile devices where low latency is paramount.
2. **Training Cost:** A significant advantage of traditional methods is their remarkably low training cost. The SVM model required less than one second for training, whereas the more complex CNN-BiGRU model’s training time was up to 55 times longer on the UniMiB SHAR dataset. The KNN classifier, requiring no training process, offers the potential for immediate deployment. This efficiency is crucial for future applications that might involve on-device model personalization.
3. **Model Size and Practical Trade-offs:** The analysis of model size highlights an important trade-off. While KNN offers the fastest inference, its reliance on storing the training data results in a considerably larger model footprint (up to 8.774 MB), which could be a constraint for devices with limited storage. In contrast, the SVM model emerges as a highly balanced and practical solution. It not only delivers inference speeds comparable to KNN but also maintains an extremely compact model size (<0.4 MB), making it an ideal candidate for resource-constrained environments.

In summary, this quantitative benchmark demonstrates that while deep learning models are powerful, our 3s2sub framework combined with traditional classifiers like SVM offers a more compelling balance of competitive detection accuracy, superior computational efficiency, and minimal resource footprint, validating its design for practical smartphone-based fall detection.

### 6.4. Evaluation on FARSEEING Dataset

As shown in former sections, our method *3s2sub* performed well on two simulated datasets in same-dataset validation. However, there may be differences between simulated fall data and real-world fall data. Therefore, we performed a cross-dataset evaluation on FARSEEING.

We perform streaming evaluation on unsegmented continuous recordings. For window length *W* and hop *H*, the decision for window t is available only at time tH+W (no look-ahead). Alarm timestamps are taken at the window end. We adopt the OR rule to prioritize sensitivity and earliest detection in safety-critical fall detection.

The experimental process is as follows:

(1) Since the data in FARSEEING were sampled at 100 Hz or 20 Hz, we processed the data to 50 Hz. For those sampled at 100 Hz, we performed down sampling. For those sampled at 20 Hz, we performed linear interpolation.

(2) After analyzing the results of the evaluation on UniMiB SHAR dataset and MobiAct dataset, we selected KNN as the classifier. Because KNN with *3s2sub* showed better scalability—it showed higher accuracy and specificity in cross-dataset validation. Although SVM showed higher sensitivity, its low specificity was unacceptable. The model was trained on the UniMiB SHAR dataset. Because it contains more types of falls and more fall samples.

(3) A sliding window (3 s) and *3s2sub* is applied to FARSEEING data to extract features. Each window is considered as an ‘event’. Overlap between windows is an important hyper-parameter. We tested overlap from 10% to 90% (10% increments).

As a result, as detailed in Table 11 our model achieved 93.35% sensitivity when the sliding window overlap was 30%. It achieved a specificity of 98.12% for all data, 98.51% for Position A and 97.28% for Position B.

## 7. Interpretability Analysis

In addition to the performance evaluations, SHAP-based [28] interpretability analysis was employed to elucidate the contribution of different features and provide insights into the decision-making process of the model.

**Figure 11:**
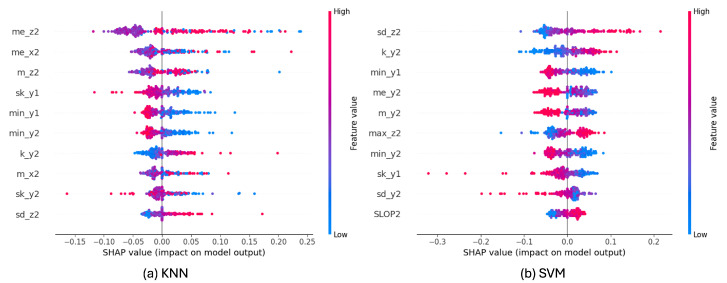
Bees warm plot of SHAP analysis.

Figure 11 shows the top-10 feature beeswarm plots on the MobiAct dataset for both KNN and SVM classifiers. For KNN (Figure 11a), the most influential features are predominantly derived from the *z*-axis and *x*-axis, such as me_z2, me_x2, and m_z2, indicating that vertical and forward-backward acceleration statistics play a critical role in fall detection. Similarly, for SVM (Figure 11b), sd_z2, k_y2, and min_y1 are among the top contributors, suggesting that variations and extremum values along the *y*- and *z*-axes are highly discriminative.

**Figure 12:**
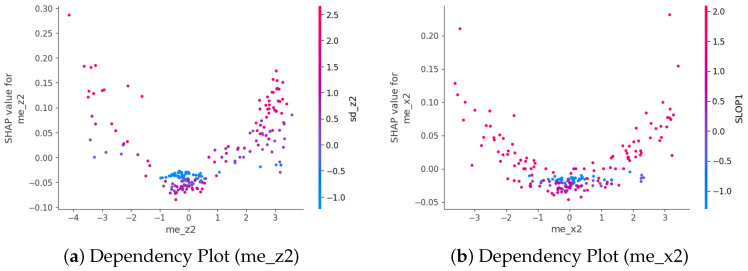
Feature Dependency(KNN).

**Figure 13:**
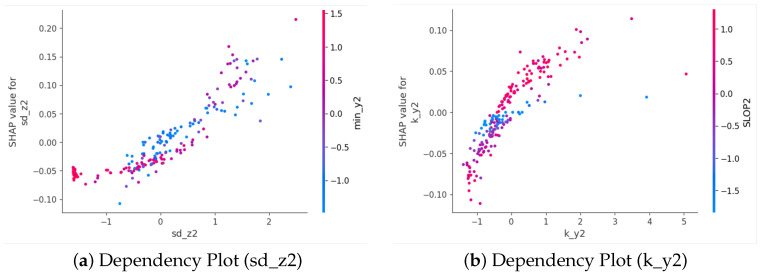
Feature Dependency(SVM).

To further explore how individual features influence predictions, feature dependency plots were generated (Figure 12 and Figure 13). We present the dependency plot of the top-ranked feature in combination with the second- and third-ranked features. For KNN, me_z2 shows the strongest interaction with sd_z2, with a clear non-linear relationship to the SHAP value. When me_z2 is positive—particularly at higher values—both sd_z2 and the SHAP value tend to increase, indicating a higher likelihood of fall classification. Similarly, me_x2 exhibits a U-shaped relationship with SHAP values, where extreme positive or negative values are associated with higher SHAP contributions, especially when SLOP1 values are high.

For SVM, sd_z2 shows a strong positive relationship with its SHAP value (Figure 13a). As sd_z2 increases, the SHAP value also rises, indicating that higher variability in the *z*-axis acceleration of the second sub-window contributes more strongly toward predicting a fall. The color gradient suggests that this effect is further modulated by min_y2, where higher values (more red) are generally associated with stronger positive SHAP contributions.

Similarly, k_y2 also exhibits a positive association with its SHAP value (Figure 13b). Larger k_y2 values tend to increase the likelihood of a fall prediction, with min_y2 again serving as a modulating factor. These results highlight the importance of kurtosis and variability features derived from the *y*- and *z*-axes in the SVM model’s decision- making process.

**Figure 14:**
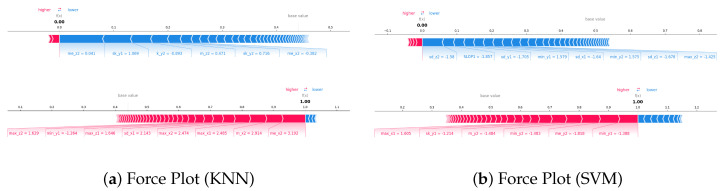
Comparison of force plots for KNN and SVM.

Finally, force plots were used to visualize feature contributions for specific instances of fall and ADL events (Figure 14). These plots reveal how individual feature values push the model output toward a fall or ADL prediction. For instance, in the KNN fall case, high values of me_x2 and m_x2 strongly increase the likelihood of predicting a fall.

For SVM, features such as k_y2 and min_y2 play a similar decisive role. This interpretive analysis confirms that the proposed method effectively captures the motion patterns most relevant to fall detection, and highlights the complementary nature of different statistical features extracted from multiple sensor axes.

## 8. Discussion

As demonstrated in the previous section, our method—*3s2sub*, achieved impressive accuracy, indicating effective fall detection capabilities.

### 8.1. Preliminary

Preliminary results provide empirical evidence supporting the design choices in the proposed *3s2sub* framework. Experiments on window length confirm that a 3 s window achieves the optimal balance between capturing the full fall dynamics and avoiding irrelevant motion. Experiments on the number of subwindows further demonstrate that partitioning the window into two overlapping parts is sufficient to reduce redundancy while maintaining temporal resolution. Finally, overlap analysis demonstrates that a 50% overlap ensures balanced coverage of the critical pre-impact and post-impact phases of the fall process. These findings validate the parameter settings adopted in our preliminary evaluation and highlight the robustness of the *3s2sub* design across different datasets and classifiers.

### 8.2. Single-Event Dataset Evaluation

In both 5-Fold and 30-Fold Cross-Validation, *3s2sub* outperformed all the control groups. Notably, there was a significant difference between *3s2sub* and the *2s1*, *2s2*, and *Raw Data* groups; *3s2sub* was considered the most effective among them. Here, we attempt to prove whether separating the window into two sub-windows makes sense or not. In Figure 5, Figure 6, Figure 8, and Figure 9, although there is no significant difference between *3s2sub* and *3s*, *3s2sub* has shorter boxes, meaning the test result distribution is more concentrated, so we believe that *3s2sub* is more stable than *3s* in these cases. In practice, we hope to reduce user operation; in other words, we hope the FDS does not need to collect data from users and be further retrained. As indicated by the results of the LOSO Cross-Validation, there is a significant difference between *3s2sub* and *3s* at the 0.1% level, so we believe that *3s2sub* can detect falls from unseen users more effectively. Additionally, the result distribution of *3s2sub* is more concentrated, so it is more stable. Overall, comparing the results between *3s2sub* and *3s*, we can conclude that *3s2sub* is more effective than *3s*. Thus, it makes sense to divide the window into two sub-windows. Beyond *3s2sub*, we have tested *3s3sub* (divide the 3 s window into three 1.5 s sub-windows and set the overlap between adjacent sub-windows to 50%). The results are not shown, because in 5-fold, 30-fold, and LOSO cross-validation there is no significant difference in T-test. Thus, we believe it is not necessary to divide the whole window into three sub-windows because it increases computational complexity.

As shown in Table 4, Table 5 and Table 6, *3s2sub* achieved the highest sensitivity, specificity, and F1 score in 5-Fold and 30-Fold Cross-Validation among all groups. In LOSO Cross-Validation, *3s2sub* achieved the highest sensitivity, precision, and F1 score with SVM. For the test with KNN, it achieved the highest sensitivity and F1 score. The specificity of *3s2sub* (98.31%) was slightly lower than that of the *Raw Data* group (98.94%). This might be a flaw, but it is still comparable. The precision and F1 score of *Raw Data* (92.59% and 95.60%) were obviously lower than those of *3s2sub* (97.59% and 97.89%). In addition, the accuracy and MAA of *Raw Data* were also lower than those of *3s2sub*. Thus, taking various indicators into account, we believe that *3s2sub* showed an advantage.

We performed an evaluation with the KNN and SVM classifiers. For the single-event data evaluation, as shown in Table 4, Table 5 and Table 6, in the non-subject-difference (5-Fold and 30-Fold) cross-validation, KNN showed higher specificity and F1 score, while SVM showed higher sensitivity. In LOSO cross-validation, SVM showed higher precision and F1 score; KNN showed slightly higher specificity as well, but the difference was less than 1%. Overall, for single-event dataset evaluation, KNN and SVM showed similar performance.

### 8.3. Continuous-Event Dataset Evaluation

In the evaluation on the MobiAct dataset, as shown in Table 7, in same-dataset validation, SVM and KNN exhibit similar performance levels in same-dataset validation. All evaluation metrics are at a similar level with those on UniMiB SHAR. KNN and SVM perform similarly, SVM shows slightly higher sensitivity while KNN shows higher specificity. These results are consistent with those on UniMiB SHAR.

In cross-dataset validation on UniMiB SHAR and MobiAct, results shown in Table 8 indicate that KNN significantly outperforms SVM in this evaluation. Concretely, SVM is more sensitive (5% higher than KNN) for fall events, which is consistent with same-dataset evaluation (both on UniMiB SHAR and MobiAct), but accuracy (8% lower), F1 score (13% lower), and specificity (12% lower) are worse than KNN. The low specificity is unacceptable in a practical FDS. In addition, compared with results of same-dataset validation (shown in Table 7), the performance of SVM drops significantly while KNN appears to be more resilient. Although the performance of KNN drops slightly, it remains acceptable. We believe this is a reasonable descent because of the difference between two datasets.

We attribute this performance gap primarily to domain shift between the training (UniMiB SHAR) and testing (MobiAct) datasets, which manifests in two major forms:

1. Subject domain shift—Motion patterns vary across individuals even for the same activity. This is evident even within a single dataset: as shown in Table 5, Table 6 and Table 7, Leave-One-Subject-Out (LOSO) cross-validation consistently produces lower accuracy than random 30-fold validation, highlighting the impact of inter-subject variability.
2. Activity domain shift—The activity definitions and distributions differ substantially between UniMiB SHAR and MobiAct. UniMiB SHAR contains short-duration, well-segmented activities such as “Sitting down,” “Jumping,” and “Syncope,” while MobiAct includes more transitional activities which are not contained in UniMiB SHAR, such as “Step into a car” (CSI), “Step out of a car” (CSO).

Interestingly, while SVM appears more robust to subject-level variation—exhibiting a smaller drop from 30-fold to LOSO within the same dataset—it performs considerably worse than KNN in the cross-dataset scenario. This suggests that SVM may be more sensitive to activity-level domain shift, while KNN offers greater robustness under heterogeneous activity structures and execution contexts.

Based on this analysis, we selected the *3s2sub* method with the KNN classifier for subsequent cross-dataset evaluation on the FARSEEING dataset, as it provides a better trade-off between sensitivity and generalization under domain shift conditions.

Table 12 shows a brief comparison of some smartphone-based fall detection algorithms, including two works that evaluated using UniMiB SHAR and two using MobiAct. It can be seen that our method achieved comparable results with state-of-the-art works, while using fewer vector, which indicates less computation.

In addition, we performed cross-dataset evaluation on the FARSEEING dataset, which is a long-term (1200 s) real-world fall dataset. It included data collected from 15 subjects. Although FARSEEING data were not collected by smartphones, and device positions were not completely same as in our training set (UniMiB SHAR), we still used it to evaluate our method. Because (1) there may be differences between real-world data and lab-simulated data, it is necessary to evaluate using real-world data; (2) the sampling range (±2g or ±6g) is the same as the smartphone’s built-in accelerometer; (3) the two positions in FARSEEING can be considered as two cases where the smartphone is placed in a very shallow trousers pocket (Position A) and a very deep trousers pocket (Position B). As the results shown in Table 11, our method achieved 95.45% sensitivity (1 sample failed to detect) and 98.12% specificity on data from both positions, with 20% overlap of the sliding window. Concretely, for data collected from Position A, our model achieved a specificity of 98.51%, while for data collected from Position B, the specificity was 97.28%.

We think the reason for this difference is that Position A is closer to the position of a normal trousers pocket, and it is closer to the center of gravity of the human body, for which the data at this position are more suitable for measuring the overall movement of the human body, while Position B is too low. The data collected at this position may contain more noise caused by leg-only movements.

**Figure 15:**
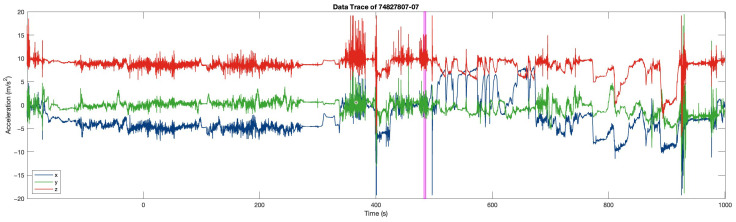
An example data trace in FARSEEING. Magenta rectangular is the true positive window.

**Figure 16:**
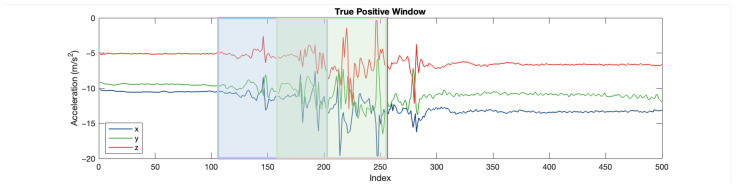
Data trace excerpt around true positive window.

Figure 15 is an example data trace in FARSEEING. The magenta rectangular is the true positive (fall) window detected. Figure 16 is the excerpt around the true positive window (for 10 s). The blue and green rectangular represents the two sub-windows. In the first sub-window (in blue), the acceleration changes obviously, with an amplitude of about ±0.5g, but minor than that in the second sub-window. In the second sub-window (about ±1g). This difference is consistent with our experience. The acceleration increases at the beginning of the fall, the speed of the body increases during the fall, and the speed becomes 0 at the moment of hitting the ground (the end of the fall), resulting in a greater acceleration at the end of the fall.

**Figure 17:**
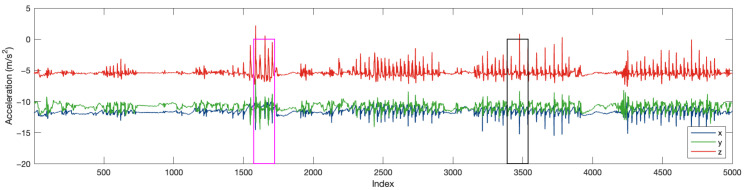
Data trace excerpt around false positive window.

Figure 17 shows the data trace excerpt around false positive of the sample failed to detect.

Table 13 summarizes the works that performed cross-dataset validation on FARSEEING. J. Silva et al. [32] proposed a transfer learning-based approach for FDS. They set 7.5 s window without overlap. With training on a self collected simulated dataset and test on FARSEEING, their method achieved 92% sensitivity. A. K. Bourke et al. [33] proposed a ML based fall detection method that used Decision Tree (DT) as the classifier. They used a more complete version of FARSEEING, including 89 falls and 368 ADLs, which are all collected at Position A to evaluate the model. They reached accuracy of 88% and specificity of 87%. Yu, X. [34] proposed the KFall dataset. They collected data at Position A with an inertial sensor. They used KFall to train a ConvLSTM model and tested it on FARSEEING. They achieved sensitivity of 93.33% and 73.33% specificity. Koo, B. [35] proposed a Neural Network based approach which is called TinyFallNet. They used the KFall [34] to train and achieved 86.67% sensitivity.

As shown, we achieved comparable sensitivity and specificity among these works.

Evaluation on the FARSEEING dataset is a key test of our approach, as it addresses the well-known “simulation-to-real world” gap faced by many fall detection systems. The scarcity of large-scale, publicly available real-world fall datasets is a major bottleneck in the research community, making any evaluation on such data, regardless of scale, extremely valuable.

However, we must acknowledge a key limitation of the FARSEEING dataset: its small sample size of only 22 verified fall events. This requires careful interpretation of the results, as it limits their broad statistical reliability. Therefore, our discussion of these results focuses not on claims of absolute real-world superiority, but rather on how this successful cross-dataset validation reveals the robustness and generalization capabilities of our 3s2sub feature set.

Despite being trained exclusively on simulated, laboratory-collected data (UniMiB SHAR), our model performs remarkably well on unseen, noisy, and heterogeneous real-world data such as FARSEEING. As shown in Table 13, our method achieves the highest reported sensitivity (95.35%) and specificity (98.12%) compared to other published studies using this challenging dataset. The key to this result lies not only in the high scores themselves, but also in the fact that our feature engineering approach successfully transfers from the simulated to the real-world domain. This demonstrates that the statistical features we designed effectively capture the fundamentally invariant physical patterns of fall events, making them less susceptible to domain shifts that can degrade the performance of other models.

In summary, despite the limitations of the evaluation on FARSEEING, it provides strong evidence for the robustness of the 3s2sub framework. It demonstrates that a well-designed feature-based approach can achieve generalization from simulation to the real world, a critical and often difficult requirement for practical fall detection systems. We believe the success in such data-scarce conditions can be attributed to several factors: (1) the discriminative power of the proposed 3s2sub feature extraction method; (2) the resampling strategy to counteract class imbalance; and (3) the model’s reliance on generalizable, interpretable features, as confirmed by the SHAP analysis. Overall, these results indicate that the proposed approach can robustly handle both class imbalance and few-shot learning challenges in fall detection, making it well-suited for deployment in real-world settings where fall data is limited and imbalanced.

### 8.4. Interpretability Analysis

SHAP-based interpretability analysis clarifies how the model utilizes the extracted features to make decisions. Bees warm plot revealed top-10 features. For KNN and SVM classifier, the features contribute differently. Dependency graph analysis revealed strong feature interactions—for example, between me_z2 and sd_z2, me_x2 and SLOP1 in the KNN model, and between sd_z2 and min_y2, k_y2 and SLOP2 in the SVM model—indicating that the combined effects of variability and central tendency in specific motion directions are crucial for distinguishing falls from ADLs.

Force graph visualization confirmed that these features work synergistically, pushing predictions toward the fall or no-fall category, providing clear evidence for the model’s decision boundary.

Compared to related studies, our approach not only achieves competitive or superior accuracy in both same-dataset and cross-dataset settings but also provides interpretability, a feature rarely addressed in smartphone-based fall detection research. By combining performance evaluation with interpretability analysis, our work helps understand the reasons for the success of certain feature engineering and parameterization strategies, laying the foundation for future applications of the *3s2sub* framework to other activity recognition or health monitoring applications.

### 8.5. Failue Case Analysis

Our approach achieved impressive results. To provide a deeper understanding of our method and its practical feasibility, we analyzed false positives and false negatives. This analysis sheds light on some of the model’s limitations. Our analysis shows that the majority of false positives are generated by specific activities of daily living (ADLs) that involve rapid postural changes and sudden decelerations, closely resembling the characteristics of real falls. In the UniMiB SHAR dataset, the most frequently misclassified ADLs are “sitting down” and “falling from a standing position to lying down.” In the MobiAct dataset, “car stepping on” (CSI) is the primary source of false positives. The underlying physical reason for these misclassifications is that these activities produce sharp, high-intensity acceleration spikes upon impact (for example, with a chair or bed), which our model can confuse with the impact phase of a fall. This is a classic challenge in the field, and our analysis accurately identifies specific ADLs that require more sophisticated feature differentiation in future work.

Our investigation of false positives reveals that missed falls are primarily atypical or complex events, with acceleration profiles that differ from standard hard-hit falls. In the UniMiB SHAR dataset, missed falls include events such as “fainting,” “falling into an obstacle,” and “falling backward into a chair.” Similarly, in the MobiAct dataset, events such as “falling backward into a chair” (BSC) and “falling forward onto one’s knees” (FKL) are sometimes missed. These events share a common characteristic: non-standard impact features. For example, a fall cushioned by a chair (BSC) results in significantly reduced peak acceleration. Falls caused by syncope may manifest as a slow slip rather than a rapid fall. These findings are crucial because they highlight the model’s limitations with soft-impact or multi-stage impact falls and indicate the need for adding features sensitive to more subtle fall patterns.

In addition, Figure 17 shows the data trace for the only false negative example on FARSEEING. We observe that the sensor recording range for this particular sample is ±6g, which differs from the ±2g range of our entire training set. This failure highlights the challenges posed by heterogeneity in real-world data, such as variations in sensor hardware. It emphasizes that, while this model is already strong, its robustness can be further improved by incorporating more diverse data during training.

## 9. Conclusions and Future Work

In this work, we proposed a smartphone-based fall detection algorithm, named *3s2sub*, which leverages accelerometer data collected from a built-in smartphone sensor. The algorithm assumes a motion event duration of 3 s (window size) and divides each window into two overlapping sub-windows to extract eight types of statistical features, resulting in a 44-dimensional feature vector for each event. This vector is then fed into a machine learning classifier to determine whether a fall has occurred.

Preliminary experiments quantified the impact of key design parameters, including window length, sub-window number, and overlap ratio, confirming that the chosen configuration (3 s window, two sub-windows, 50% overlap) provides the optimal balance between temporal coverage and discriminative power.

We evaluated the proposed method on three datasets: UniMiB SHAR (single-event), MobiAct, and FARSEEING (both continuous-event). UniMiB SHAR and MobiAct are laboratory-simulated datasets, while FARSEEING contains real-world fall events. Same-dataset validation on UniMiB SHAR and MobiAct yielded performance comparable to state-of-the-art methods. In cross-dataset validation—training on UniMiB SHAR and testing on MobiAct and FARSEEING—our model achieved outstanding results, including 95.31% sensitivity and 98.12% specificity, the highest among similar works. These results confirm the effectiveness and robustness of the *3s2sub* approach for fall detection.

Furthermore, SHAP-based interpretability analysis further revealed that the most influential features are primarily derived from the *z*-axis and *x*-axis accelerations, with strong interactions between statistical measures such as mean, standard deviation, and kurtosis. These insights not only validate the rationale behind the *3s2sub* design but also enhance the transparency of the model’s decision-making process.

This study focuses on algorithmic design and does not include an on-device app; consequently, device-specific I/O, OS scheduling/background execution, and energy/thermal behavior are out of scope. We view a system-level prototype with end-to-end latency and energy profiling as an important avenue for future work.

There are some future directions. (1) Power consumption: Power consumption is a key consideration for smartphone-based methods. While we did not address this in the current study, as all experiments were conducted on a computer, it will be an important factor for future work. Power consumption is influenced by many factors, including system design and configuration, and will be a focus in the development of practical applications. (2) System architecture optimization: To develop a practical and user-friendly system, we will enhance the system architecture. This includes optimizing data storage and improving communication with cloud or fog nodes to support more efficient data processing and decision-making. (3) Real-World data collection: In this study, we utilized three datasets—UniMiB SHAR, MobiAct, and FARSEEING. The first two were collected in simulated environments, while FARSEEING contains real fall events. However, the number of real falls is limited, as capturing them requires long-term monitoring, making it challenging. In the future, we aim to collect more real-world fall data to improve the robustness of our system. (4) Detailed fall detection: Our goal is to provide more detailed motion detection, such as identifying the specific type of fall. This could provide valuable information to healthcare providers about the severity and potential risk associated with the fall, enhancing the system’s utility. (5) Generalized model for smartphone positioning: So far, we have focused on a smartphone-based fall detection system where the device is placed in the user’s trousers pocket. However, smartphones can also be placed in other locations, such as a chest pocket or a bag. Developing a generalized model that accounts for different smartphone placements is a key challenge that we will address. We plan to collect motion data from different positions and combining our *3s2sub* method with ensemble learning, such as using Bagging to ensemble multiple locations equally, using Boosting for data at difficult-to-classify locations, etc.

## Figures and Tables

**Table 1:** Features.

Number	Feature
1	Mean
2	Median
3	Standard Deviation (SD)
4	Skew
5	Kurtosis
6	Maximun
7	Minimum
8	Slop (SL) [20]

**Table 2:** Average fall detection accuracy ^1^.

Classifier	Feature Vector	5-Fold	30-Fold	LOSO ^2^
KNN	*3s2sub*	**99.77%**	**99.78%**	**97.36%**
	*2s1*	99.40%	99.63%	94.58%
	*2s2*	99.13%	99.28%	94.52%
	*3s*	99.69%	99.77%	95.71%
	*Raw Data*	98.30%	98.53%	94.29%
SVM	*3s2sub*	**99.73%**	**99.74%**	**98.45%**
	*2s1*	99.37%	99.41%	97.10%
	*2s2*	98.89%	99.01%	96.18%
	*3s*	99.58%	99.62%	97.51%
	*Raw Data*	98.84%	98.95%	97.29%

^1^ Average accuracy of each control group with KNN and SVM classifier. In bold are the best results. ^2^ LOSO: Refers to Leave-One-Subject-Out Cross-Validation.

**Table 3:** Average fall detection Macro Average Accuracy (MAA) ^1^.

Classifier	Feature Vector	5-Fold	30-Fold	LOSO ^2^
KNN	*3s2sub*	**99.70%**	**99.70%**	**96.93%**
	*2s1*	99.24%	99.55%	93.68%
	*2s2*	98.90%	99.08%	93.92%
	*3s*	99.63%	99.71%	95.45%
	*Raw Data*	97.65% ^3^	97.97%	92.67% ^4^
SVM	*3s2sub*	**99.71%**	**99.72%**	**98.48%**
	*2s1*	99.28%	99.32%	97.09%
	*2s2*	98.77%	98.89%	96.33%
	*3s*	99.53%	99.58%	97.66%
	*Raw Data*	98.84% ^5^	98.96%	97.24% ^6^

^1^: Average macro average accuracy of each control group with KNN and SVM classifier. In bold are the best results. ^2^ LOSO: Refers to Leave-One-Subject-Out Cross-Validation. ^3^: Claimed 97.78% in [29]. ^4^: Claimed 92.90% in [29]. ^5^: Claimed 98.71% in [29]. ^6^: Claimed 97.57% in [29].

**Table 4:** Fall detection results (*5-Fold Cross-Validation*) ^1^.

Classifier	Evaluation Metric	3s2sub	2s1	2s2	3s	Raw Data
KNN	Specificity	**99.93%**	99.78%	99.59%	99.85%	99.89%
	Precision	**99.74%**	99.32%	98.94%	99.72%	97.57%
	F1 Score	**99.84%**	99.55%	99.26%	99.79%	98.72%
	Sensitivity	99.52%	98.76%	98.07%	99.50%	95.49%
SVM	Specificity	**99.75%**	99.50%	99.12%	99.75%	98.81%
	Precision	**99.82%**	99.38%	99.10%	99.67%	99.40%
	F1 Score	**99.78%**	99.44%	99.11%	99.71%	99.11%
	Sensitivity	99.67%	98.88%	98.38%	99.40%	98.93%

^1^ Average sensitivity, precision and F1 score of all tested groups with KNN and SVM classifier, in 5-Fold Cross-validation. In bold are the highest results of each evaluation metric, with KNN or SVM.

**Table 5:** Fall detection results (*30-Fold Cross-Validation*) ^1^.

Classifier	Evaluation Metric	3s2sub	2s1	2s2	3s	Raw Data
KNN	Sensitivity	**99.96%**	99.83%	99.76%	99.91%	99.92%
	Precision	**99.70%**	99.61%	99.12%	99.74%	97.85%
	F1 Score	**99.83%**	99.72%	99.44%	99.82%	98.88%
	Specificity	**99.45%**	99.28%	98.39%	99.52%	96.02%
SVM	Specificity	**99.78%**	99.64%	99.30%	99.74%	98.94%
	Precision	**99.82%**	99.45%	99.17%	99.68%	99.43%
	F1 Score	**99.79%**	99.54%	99.23%	99.71%	99.18%
	Sensitivity	**99.66%**	98.99%	98.49%	99.42%	98.97%

^1^ Average sensitivity, precision and F1 score of all tested groups with KNN and SVM classifier, in (non-subject-difference) 30-Fold Cross-validation. In bold are the highest results of each evaluation metric, with KNN or SVM.

**Table 6:** Fall detection results (*Leave-One-Subject-Out Cross-Validation*) ^1^.

Classifier	Evaluation Metric	3s2sub	2s1	2s2	3s	Raw Data
KNN	Specificity	98.31%	96.49%	95.85%	96.34%	**98.94%**
	Precision	**97.59%**	95.19%	95.56%	97.03%	92.59%
	F1 Score	**97.89%**	95.76%	95.55%	96.52%	95.60%
	Sensitivity	**95.55**%	90.86%	91.99%	94.55%	86.40%
SVM	Specificity	**98.15%**	96.96%	95.77%	96.92%	97.36%
	Precision	**99.35%**	98.33%	98.15%	99.07%	98.28%
	F1 Score	**98.68%**	97.59%	96.81%	97.90%	97.78%
	Sensitivity	**98.83**%	97.19%	96.92%	98.36%	97.05%

^1^ Average sensitivity, precision and F1 score of all tested groups with KNN and SVM classifier, in LOSO Cross-validation. In bold are the highest results of each evaluation metric, with KNN or SVM.

**Table 7:** MobiAct test results with models trained on MobiAct.

Classifier	Accuracy	Sensitivity	F1 Score	Specificity	Precision
KNN	**99.84%**	**98.02%**	**98.01%**	**99.92%**	**98.00%**
SVM	99.76%	96.98%	97.04%	99.88%	96.98%

**Table 8:** MobiAct test results with models trained on UniMiB SHAR.

Classifier	Accuracy	Sensitivity	F1 Score	Specificity	Precision
KNN	**96.41%**	92.05%	**92.47%**	**97.78%**	**92.47%**
SVM	88.22%	**97.26%**	79.83%	85.36%	92.05%

**Table 9:** Head-to-head comparison.

Dataset	Method	Accuracy	Sensitivity	Precision	F1 Score	Specificity
MobiAct	*3s2sub* + KNN	**99.84%**	**98.02%**	**98.00%**	**98.01%**	**99.92%**
MobiAct	*3s2sub* + SVM	99.76%	96.98%	96.98%	97.04%	99.88%
MobiAct	1D-CNN	99.49%	96.55%	97.00	96.72%	99.50%
MobiAct	CNN-BiGRU [25]	99.44%	97.20	97.50	97.34%	99.34%
UniMiB SHAR	*3s2sub* + KNN	**99.83%**	**100%**	**99.74%**	**99.87%**	**100%**
UniMiB SHAR	*3s2sub* + SVM	99.24%	99.21%	99.60%	99.40%	99.21%
UniMiB SHAR	1D-CNN	96.64%	96.46%	94.29%	95.36%	96.75%
UniMiB SHAR	CNN-BiGRU [25]	97.82%	97.39%	96.54%	96.96%	98.06%

**Table 10:** Computational analysis.

Dataset	Method	Training Time	Inference Time (per Window)	Model Size (MB)
MobiAct	SVM	0.53 s	**0.0332 ms**	0.1807
MobiAct	KNN	— ^1^	0.0443 ms	8.774
MobiAct	1D-CNN	2.036 s	0.16 ms	0.1837
MobiAct	CNN-BiGRU	5.175 s	0.2 ms	3.473
UniMiB SHAR	SVM	0.40 s	0.069 ms	0.3939
UniMiB SHAR	KNN	— ^1^	**0.015 ms**	4.685
UniMiB SHAR	1D-CNN	2.752 s	0.20 ms	0.1793
UniMiB SHAR	CNN-BiGRU	22.261 s	0.96 ms	2.164

^1^ KNN does not have a training process.

**Table 11:** FARSEEING streaming protocol test results with model trained on UniMiB SHAR (30% overlap).

Sensor Position	Sensitivity	Specificity
A	93.33% (14/15)	98.51%
B	100%	97.28%
A & B	95.35%	98.12%

**Table 12:** Comparison of smartphone-based FDS (results on simulated fall datasets).

Work	Sensor	Model/Classifier	Feature Amount	Accuracy	Sensitivity	F1 Score	Precision
[9]	Accelerometer	Linear classifier	NS	99.7%	96.3%	97.7%	99.2%
[10]	Accelerometer, Gyroscope	BDT	44	99.9%	NS	NS	NS
[12]	Accelerometer	Threshold	3 Thresholds	99.38%	NS	NS	NS
[13]	Accelerometer	SVM with a newly proposed kernel	5	99.25%	100%	99.53%	98.12%
[14] ^2^	Accelerometer, Gyroscope, Magnetometer, Gravity, Linear acceleration	SVM, KNN, Decision Tree, Random Forest, Naive Bayes	45	95.65%	NS	NS	NS
[19] ^4^	Accelerometer, Gyroscope, Orientation Sensor	CNN-LSTM	58	96.75%	95%	97%	97%
[22] ^4^	Accelerometer	GRU	8	99.39%	NS ^1^	95.06%	96.69%
[24] ^4^	Accelerometer	EnsemConvNet	-	99.7%	NS	NS	NS
[25]	Accelerometer	PCNN-Transformer	-	98.30%	98.99%	97.91%	98.90%
[26]	Accelerometer	FFNN	-	99.38%	99.79%	99.24%	98.69%
[27]	Accelerometer	MKLS-Net	-	99.40%	99.23%	99.40%	99.40%
[29] ^3^	Accelerometer	SVM	151	98.71%	NS	NS	NS
**This Work** ^3^	Accelerometer	SVM, KNN	44	99.77%	99.96%	99.84%	99.82%
**This Work** ^4^	Accelerometer	SVM, KNN	44	99.89%	98.67%	98.67%	98.00%

^1^ NS: Not specified. ^2^ Here shows the highest accuracy claimed, by gyroscope and SVM. ^3^ Used UniMiB SHAR to evaluate. ^4^ Used MobiAct to evaluate.

**Table 13:** Comparison of Performances between Past Models and Proposed *3s2sub* Model on Real-World Fall Data (FARSEEING).

Work (Year)	Model	Sensitivity	Specificity
[32] (2018)	MLP	92%	NS
[33] (2016)	Decision Tree ^1^	88%	87%
[35] (2023)	TinyFallNet	86.67%	NS
[34] (2021)	ConvLSTM	93.33%	73.33%
**This Work (2025)**	KNN	**95.35% (21/22)**	**98.12%** (**98.51%** for Position A, **97.28%** for Position B)

^1^: In this work, authors used full version FARSEEING datasets including 89 Fall samples.

## Data Availability

In this paper, we used public datasets UniMiB SHAR, MobiAct and FARSEEING to perform experiments. They are available from the following URLs: UniMiB SHAR—http://www.sal.disco.unimib.it/technologies/unimib-shar/, accessed on 1 October 2021. MobiAct—https://bmi.hmu.gr/the-mobifall-and-mobiact-datasets-2/, accessed on 1 October 2022. FARSEEING—https://www.ehtel.eu/activities/eu-funded-projects/farseeing.html, accessed on 1 June 2024. Note: The official FARSEEING dataset website is currently unavailable (as of 22 October 2025). The dataset was obtained through direct contact with the project’s corresponding author, as the online access link provided by the project is no longer active.
